# The Association between Diet–Exercise Patterns and Cirrhosis: A Cross-Sectional Study from NHANES 2017-March 2020

**DOI:** 10.3390/nu16111617

**Published:** 2024-05-25

**Authors:** Jialu Liu, Xinhao Han, Lu Chen, Liudan Mai, Xiaoman Su, Yanlin Dong, Baolong Wang, Qiuju Zhang

**Affiliations:** Department of Biostatistics, School of Public Health, Harbin Medical University, Harbin 150088, China; liu18204601667@163.com (J.L.); hmuhanxh@163.com (X.H.); nanjie0413@163.com (L.C.); liudanmai0102@163.com (L.M.); suxiaoman_eve@163.com (X.S.); dongyanlin612@163.com (Y.D.); 18866614080@163.com (B.W.)

**Keywords:** cirrhosis, dietary intake, exercise, NHANES

## Abstract

Background: Liver cirrhosis (LC) is one of the most significant causes of morbidity and mortality in patients with chronic liver disease worldwide. Nutrition may be an important component of primary prevention of chronic liver disease. Diet–exercise patterns frame the eating behaviors and exercise habits of people through statistical methods related to nutritional epidemiology, which can explore the relationship between living habits and diseases among diverse populations. The purpose of this study was to explore the association between diet–exercise patterns and cirrhosis, and provide guidance on preventive diets for liver patients. Methods: This study identified diet–exercise patterns via clustering analysis of principal components and assessed their association with cirrhosis through the population samples of the National Health and Nutrition Examination Survey (NHANES) from 2017 to March 2020. Results: We identified two diet–exercise patterns that were named the “prudent pattern” (consumption of various staple foods, eggs, meat, fruits and vegetables; less sedentary) and the “dangerous pattern” (higher consumption of desserts, nuts, milk, meat, alcoholic beverages; recreational activities). The *t*-test demonstrated a significant relationship between patterns and multiple foods. The simple logistic regression test showed a lower risk of cirrhosis in those in the “prudent pattern” (OR = 0.73, 95%CI = 0.59–0.93). Conclusions: Two diet–exercise patterns associated with cirrhosis were identified: “prudent pattern” and “dangerous pattern”. The results of this study may be useful for suggesting preventive diets for people at risk of cirrhosis.

## 1. Introduction

Liver cirrhosis is the terminal stage of most chronic liver diseases (CLDs), having the highest incidence and mortality among liver-related diseases, which has an inferior prognosis [1,2,3]. In 2019, LC was associated with 2.4% of global deaths, and cirrhosis deaths are predicted to increase over the next decade, with the rate gradually increasing in some countries [4,5]. Steatosis is associated with the progression of liver fibrosis and cirrhosis, which is the process of diffuse liver damage [6,7]. Simple steatosis (NAFL) and nonalcoholic steatohepatitis (NASH) are closely related to a series of liver injuries caused by lipotoxicity, oxidative stress and other factors [8]. Biopsy studies have proved that NAFL can progress to advanced fibrosis [9]. It eventually leads to liver regeneration and fibrogenesis, enhances collagen and extracellular matrix deposition, and gradually progresses to cirrhosis [6,10]. Cirrhosis gives rise to numerous systemic complications, including ascites, malnutrition, sarcopenia and various others [3,5,11]. Malnutrition is a prominent characteristic and a significant complication of cirrhosis. Accumulating data have suggested that patients with LC often develop protein–energy malnutrition (PEM) at a rate of 25.1–65.5%, and PEM plays a crucial role in their poor survival [12]. Therefore, there is a need for more interventions to promote primary prevention, early detection and early treatment for liver disease-related patients [4].

Risk factors for LC include age, gender, obesity, smoking, alcohol consumption, genetics, the dysregulation of gut microbes, etc. [13]. Previous studies have demonstrated that sedentary behavior is also a risk factor for fibrosis progression in chronic liver diseases. In addition, cirrhosis is associated with significant nutritional risks. Assessing the impact of a single food, a food group or a nutrient on diseases is often not conclusive, because overall dietary habits of people include a variety of foods and the components of the diet are closely intertwined [14,15]. Dietary pattern analysis, of which common methods include index analysis, factor analysis and so on, is an alternative and complementary approach to analyze the relationship between nutrition and chronic disease risk, with the advantage of taking into account possible correlations between foods and food groups, as well as between nutrients [15]. Therefore, dietary pattern analysis is a better choice to capture the complex interactions between nutrition, food and other relevant factors.

Thus, a variety of food items were included in this analysis, and we further added exercise items to increase the accuracy of the clustering. Through an unsupervised machine learning approach, we explored the diet–exercise patterns associated with cirrhosis, and assessed the association between the patterns and cirrhosis. Finally, we discussed these results, drew conclusions based on the analyses we performed and provided guidance on preventive diets and activities for liver disease patients.

## 2. Materials and Methods

### 2.1. Study Population

The data from the National Health and Nutrition Examination Survey (NHANES) from 2017 to March 2020 were used for this study. The NHANES database is a population-based national survey that was published by the National Center for Health Statistics (NCHS), US Centers for Disease Control and Prevention (CDC). It obtains a nationally representative sample of approximately 5000 individuals per year through a complex, multi-stage sampling design and updates the database every 2 years, which can be used to assess the health and nutritional status of the United States population through information from questionnaires, physical examinations and laboratory tests [16,17]. And the NHANES protocol was reviewed and approved by the National Center for Health Statistics Research Ethics Review Board [18]. All relative data are publicly available at www.cdc.gov/nchs/nhanes/, accessed on 5 September 2023.

### 2.2. Outcome Indexes

Outcome indexes: The Mobile Examination Center (MEC) performed vibration-controlled transient elastography (VCTE) on the study population using the FibroScan Model 502 V2 Touch (Echosens, Paris, France) equipped with medium (M) and extra-large (XL) probes [19]. VCTE is a widely used clinical method for evaluating hepatic steatosis and fibrosis through controlled attenuation parameters (CAPs) and liver stiffness measurements (LSMs), respectively, obtained by measuring the shear wave velocities in liver tissue [19,20]. According to Tsochatzis et al. [21], VCTE recognizes cirrhosis with a high overall sensitivity and specificity (0.83 and 0.89, respectively). Sasso et al. [22] showed that using CAPs was a non-invasive, immediate, objective and effective method for the detection and quantification of steatosis, and can effectively separate several steatosis grades (compared with grade 1, AUC = 0.89, 0.91 and 0.95). Eddowes et al. [23] found that CAPs and LSMs can evaluate hepatic steatosis and fibrosis, respectively, with AUROC values ranging from 0.7 to 0.89. For VCTE results, examinations were considered reliable only if at least 10 LSM values were obtained after fasting for at least 3 h, with an interquartile range (IQR)/median < 30% [24].

Diagnostic criteria: We adopted the recent findings of Eddowes et al. [23] and Ciardullo et al. [25] as the disease grading criteria in this study (Table 1). Median CAP values and median LSM values were, respectively, divided into four grades.

Auxiliary indexes: Through examination and laboratory testing, the measurements of alanine aminotransferase (ALT), aspartate aminotransferase (AST), gamma-glutamyltransferase (GGT) and alkaline phosphatase (ALP) were obtained, and the NAFLD-4 (FIB-4) index and AST-to-platelet ratio index (APRI) [26] were calculated. The calculation formula was as follows: FIB-4 = (age×AST level)/(platelet count×ALT^1/2^). Cirrhosis/severe fibrosis was defined as FIB-4 > 3.25 [27]. APRI = (AST level/AST upper limit of normal) × 100/platelet count. Cirrhosis was defined as APRI > 2 [28].

### 2.3. Assessing Diet and Exercise and Constructing Patterns

In the NHANES, dietary and nutrient intake were assessed by reliable 24 h dietary recall interviews [29]. The data obtained from the 24 h recall were used to estimate individual characteristics of nutrient intake according to the Food and Nutrient Database for Dietary Studies (FNDDS) supplied by the US Department of Agriculture (USDA). The FNDDS contains a variety of food and beverage items and is updated biannually [30]. Dietary studies of the FNDDS database were used to encode and analyze dietary intakes collected in the WWEIA portion of the NHANES survey [31].

Physical activity was measured by using the global physical activity questionnaire that includes questions about frequency, intensity and duration [30]. To assess physical activities, we calculated the weekly metabolic equivalent (MET) score for each activity and intensity of the participants, referring to the methodology recommended by the NHANES [16]. With reference to the FNDDS database data, we divided all of the original food items into 53 food groups and 6 exercise groups (Table S1).

Dietary pattern analysis is an alternative and complementary approach to analyzing the relationship between nutrition and chronic disease. To determine dietary patterns, we can use priori approaches (i.e., indices or scores) to assess the conformity of diets with nutrition guidelines or established patterns. However, most analyses use posterior methods at present, such as cluster analysis and factor analysis based on exploratory data [32]. To obtain different dietary patterns, this study applied a new method developed by Maugeri et al. [33], which was defined as clustering of principal components. The method includes two multivariate techniques that both reduce the dimension of the diet datasets and provide a clustering solution [34]. Principal component analysis (PCA) is applied to reduce the data dimension of diet–exercise datasets. As a dimension reduction method commonly used in various fields including nutritional epidemiology, PCA usually selects principal components based on a cumulative variance greater than 80% or an eigenvalue greater than 1. However, for dietary data, the percentage of variance explained is typically between 10% and 30% [33]. Thus, choosing principal components based on eigenvalues is more suitable for the diet–exercise data in this study. In this step, the number of principal components retained were selected through the eigenvalues. The absolute values of factor loads were used to determine the contribution of each category to the composition. For each principal component (PC), the factor scores were calculated as the sum of the products between energy intakes and factor loads. We then performed K-means clustering on the retained PCs. Finally, the number of clusters to be considered were selected according to the clustering elbow diagram and the silhouette coefficients diagram.

### 2.4. Other Laboratory Tests and Clinical Data

Demographic information on age, gender, race/ethnicity, education, marriage, drinking and smoking status was collected through interviewer questionnaires. Race/ethnicity was divided into Mexican American, other Hispanic, non-Hispanic White, non-Hispanic Black, non-Hispanic Asian and others [35]. Educational attainment was classified as less than high school, high school graduate, some college or AA degree, college graduate or above. Marital status was classified as married, divorced/widowed/separated or unmarried. Drinking or not depended on whether the individual had consumed any alcoholic beverages or not. Smoking was defined as having smoked at least 100 cigarettes. Hypertension, diabetes and high cholesterol levels were determined based on questionnaires [36]. Also based on the questionnaires, we obtained information on whether the participants had anemia, kidney disease, cancer, hepatitis B, hepatitis C and other diseases. Other covariates in relation to the clinical and laboratory assessments were examined by trained medical experts [16].

### 2.5. Statistical Analysis

All statistical analyses accounted for complex survey design factors of the NHANES, including sample weights, stratification, and clustering, following the NHANES analytic and reporting guidelines. We used the *t*-test and rank sum test to evaluate the relationship between the patterns and the original food, the nutrients and cirrhosis-related indicators. Chi-square tests were used to detect the relationship between patterns and population characteristics, and between patterns and other diseases. Simple logistic regression tests were used to assess the association of cirrhosis with diet–exercise patterns, age (continuous), gender, education level, marital status, BMI (continuous), smoking status (smoking and non-smoking) and alcohol consumption (drinking and non-drinking). Logistic regression results were reported as odds ratios (ORs) and 95% confidence intervals (95%CIs). All statistical tests were conducted at the significance level of α = 0.05, and *p* < 0.05 indicated statistical significance [37]. All analyses were performed by using R software, version 4.2.0.

## 3. Results

### 3.1. Study Population

We investigated the association between diet–exercise patterns and the risk of cirrhosis in 7869 participants aged 18 years or older in the NHANES database. Participants with the absence of liver elastography information, diet or exercise recall information, and other covariates were excluded. A total of 5757 participants were included in this study (Figure S1).

### 3.2. Clustering of Principal Components

**Figure 1:**
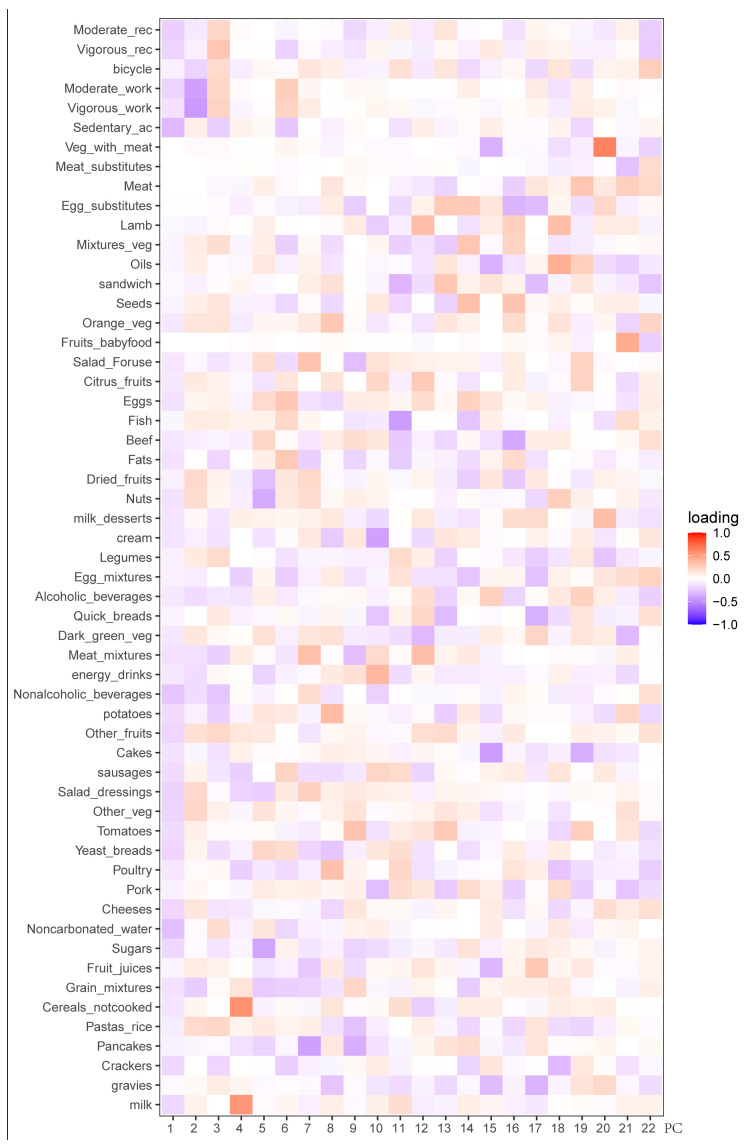
Factor loadings for each category and principal component.

We applied PCA to standardized and energy-adjusted dietary and exercise data. PCA produced 22 PCs with eigenvalues greater than 1, of which 4 PCs had eigenvalues greater than 1.2. The first 4 PCs together explained 15.27% of the total variance, and the first 22 PCs together explained 52.03% of the total variance. Figure 1 displays the factor loads related to 22 PCs. Therefore, according to the selection principle of eigenvalues greater than 1, we selected the first 22 PCs for the next step of clustering research. This choice not only greatly reduced the dimension of the original data, which was more convenient for our subsequent analysis, but also greatly retained 52.03% of the original information in the diet–exercise data, which made our clustering results more accurate in interpreting the original information. Meanwhile, we also display the features of the first four principal components so that we can observe the distribution of the original features. Table 2 and Table S2 describe the first four PC-related explanatory variances and factor loads, while Figure S2 illustrates the distribution of the samples across the principal components. PC1 was mainly characterized by less sedentary activity, but insufficient intake of water, fruits and vegetables. PC2 mainly showed a higher intake of fruit and salad dressing, as well as less engagement in work activities. PC3 mainly consumed rice and vegetables and engaged in more recreational activities. PC4 mainly consumed milk and uncooked grains.

**Figure 2:**
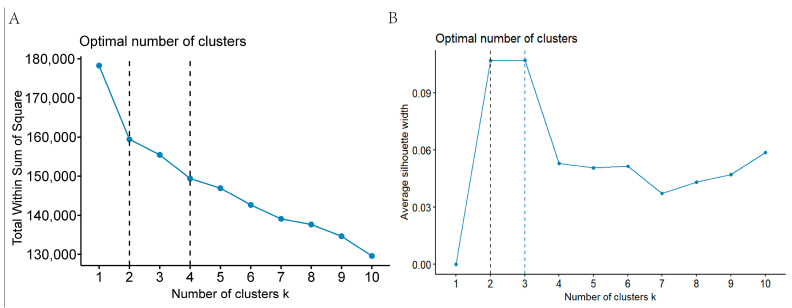
Elbow curve and silhouette scores for different cluster solutions. (**A**) Elbow curve. (**B**) Silhouette scores.

**Figure 3:**
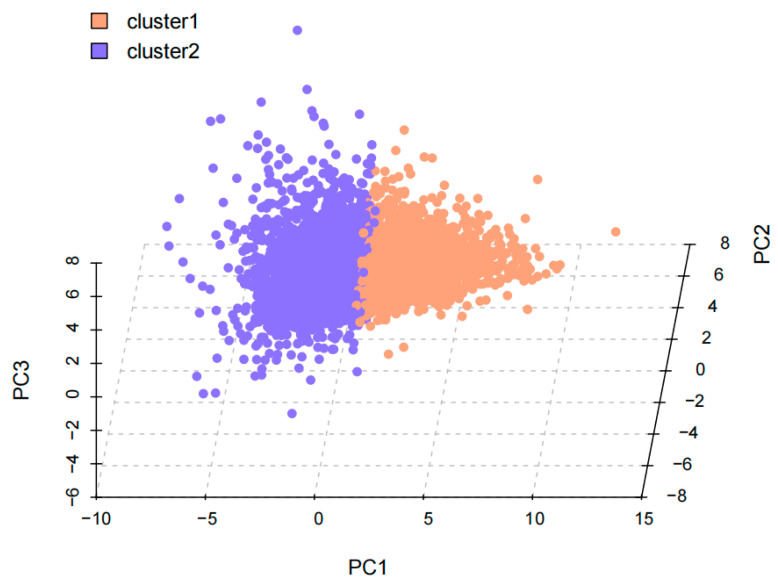
Distribution of participants by principal components and clusters.

Next, we performed clustering on 22 principal components to obtain the clustering of samples. We also drew an elbow curve of clustering and calculated the silhouette scores of different clustering solutions, choosing the solution with two clustering features (Figure 2). So, at last, we obtained two diet–exercise patterns (Figure 3).

### 3.3. Diet and Exercise Characteristics between Two Different Patterns

**Figure 4:**
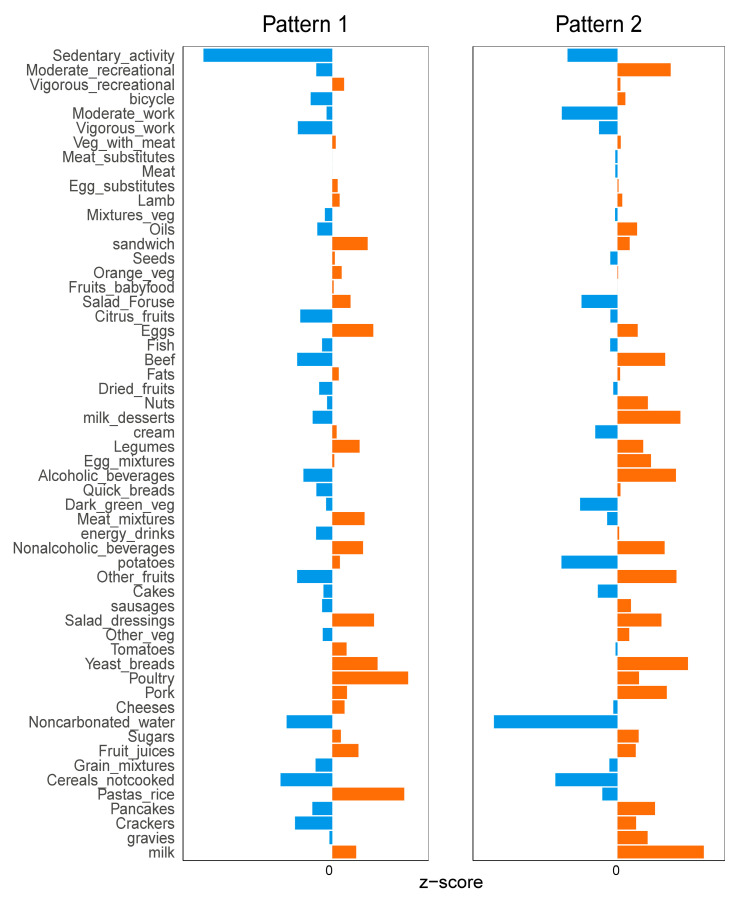
Comparison of diet–exercise items between patterns (Orange color represents a positive z-score and blue represents a negative z-score).

We compared the differences between the patterns for each diet–exercise category (Table S3). Figure 4 shows the average Z-scores for each diet–exercise category across patterns. Participants of pattern 1 consumed mostly noodles, rice, non-carbonated soda, poultry, potatoes, tomatoes, meat mixes, dark green vegetables, ice cream, fats, orange vegetables, sandwiches and lamb and tended to engage in moderate workout activities. Participants of pattern 2 were characterized by their intake of milk, cereals, biscuits, pancakes, pork, rising bread, sausages, other vegetables, other fruits, non-alcoholic beverages, alcoholic beverages, fast bread, egg mixes, desserts, nuts, beef, citrus fruits and oils, and tended to engage in moderate recreational activities and sedentary activities.

**Figure 5:**
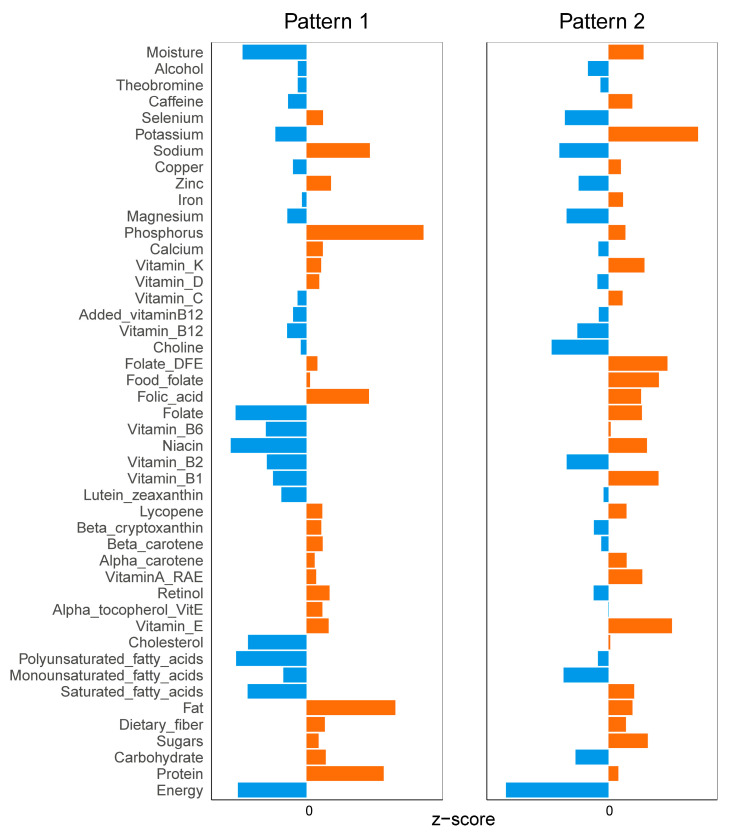
Comparison of nutrient intakes between patterns (Orange color represents a positive z-score and blue represents a negative z-score).

In terms of nutrition, participants of pattern 1 had higher intakes of protein, carbohydrates, total fat, total monounsaturated fatty acids, alpha-tocopherol vitamin E, retinol, beta-carotene, beta-zeaxanthin, lycopene, folate, vitamin B12, vitamin D, calcium, phosphorus, magnesium, zinc, sodium and selenium. Accordingly, participants of pattern 2 had higher intakes of sugar, total saturated fatty acids, total polyunsaturated fatty acids, cholesterol, vitamin E, vitamin A, alpha-carotene, vitamin B1, niacin, vitamin B6, total folic acid, dietary folic acid, vitamin C, vitamin K, iron, copper, potassium, caffeine and water (Table S4 and Figure 5).

### 3.4. Population Characteristics between Two Patterns

We then compared the basic characteristics of the two patterns of people. There were fewer people younger than 30 years in pattern 1 and more people older than 65 years in pattern 2 (*p* = 0.002). There were more women in pattern 1 (*p* < 0.001). Participants in pattern 1 were less educated (*p* < 0.001) and less often married compared to the dangerous pattern (*p* < 0.001). In addition, pattern 2 reported more participants with smoking and alcohol consumption (*p* = 0.036; *p* < 0.001). No statistically significant difference in BMI was found between the patterns (Table 3).

**Figure 6:**
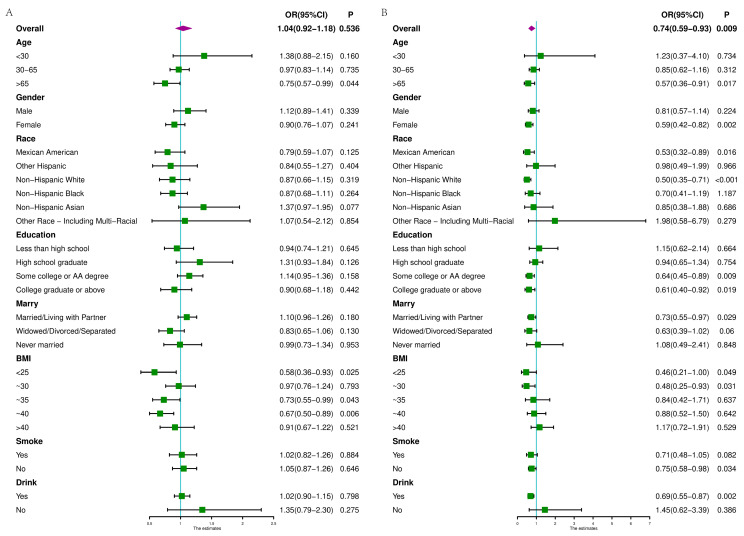
Forest plots of subgroup analyses. (**A**) Subgroup analysis between CAPs and patterns. (**B**) Subgroup analysis between LSMs and patterns.

We then analyzed how diseases were related to diet–exercise habits in different demographic subgroups. We found that although there was no significant relationship between CAPs and patterns in the overall population, there was a connection between the hepatic steatosis degree and diet–exercise patterns among people older than 65 years, people with a BMI under 25 and people with BMI of 30~40. In the overall population, the LSM was associated with the patterns. Moreover, the association between LSMs and diet–exercise patterns varied across demographic variables. For example, diet–exercise patterns had a significant effect on LSMs in people above 65 years old. However, in people under 65 years old, diet–exercise patterns could not be considered to have a significant effect on LSMs (Figure 6). We think that those with unhealthy diet–exercise habits may be more likely to suffer from diseases.

### 3.5. Risk Factors of CAPs

Next, we assessed the main factors associated with CAPs (Table 4). According to our single-factor analysis, the risk factors associated with CAPs were age, gender, race, education, marital status and BMI. Our multi-factor analysis of these factors also confirmed these results (stepwise according to AIC). At the same time, our analysis showed that hypertension, diabetes, high cholesterol level and hepatitis B were all important influencing factors for hepatic steatosis in the US population.

### 3.6. Risk Factors of LSMs

The results of univariate and multivariate analyses of LSMs are shown in Table 5. For the cluster and sociodemographic characteristics, the main factors associated with LSMs were diet–exercise patterns, age, gender, education level, BMI and smoking. People belonging to the first cluster had a lower probability of liver fibrosis and cirrhosis than those belonging to the second cluster (OR = 0.73; 95%CI = 0.59–0.93; *p* = 0.003). The diet–exercise patterns of these two clusters were named “prudent pattern” and “dangerous pattern” based on the risk of cirrhosis and the diet–exercise preferences. In addition, the risk of cirrhosis was higher in people with a higher BMI level (OR = 1.13; 95%CI = 1.11–1.15; *p* < 0.001). Hypertension, diabetes, high cholesterol level and hepatitis C were also important influencing factors for liver fibrosis and cirrhosis. We conducted a multi-factor analysis with a stepwise selection of cluster and sociodemographic characteristics, which obtained results that were almost consistent with the single-factor analysis. However, the results of the multi-factor analysis of cluster characteristics, sociodemographic characteristics and related disease characteristics showed that the influence of cluster characteristics on LSMs was no longer statistically significant.

To further support our results, we compared the differences in liver-related indicators between different patterns. ALT, AST, GGT, ALP and FIB4 scores showed statistically significant differences between the two patterns (Table 6). The FIB4 index (OR = 0.911; 95%CI = 0.885–0.937; *p* < 0.001) and APRI index (OR = 0.996; 95%CI = 0.993–1.00; *p* < 0.001) confirmed the relationship between cirrhosis and the two patterns. Specifically, the prudent pattern was found to be less likely to develop cirrhosis compared to the dangerous pattern.

Thus, we believe that diet–exercise pattern is an important influencing factor of liver cirrhosis. However, the reason for the difference between the single-factor analysis and the multi-factor analysis requires further analysis.

### 3.7. Other Diseases with Different Patterns

Table 7 shows the association between the two diet–exercise patterns and other diseases, indicating that there are significant differences in the prevalence of hypertension, diabetes and anemia between the two patterns (*p* < 0.001), and high cholesterol levels are also associated with the two diet–exercise patterns (*p* = 0.036). Since hypertension, diabetes and high cholesterol levels are important influencing factors of liver cirrhosis and closely related to the diet–exercise patterns, we analyzed whether the role of diet–exercise patterns in cirrhosis may be influenced by these diseases.

### 3.8. The Medication Effect of the Disease

**Figure 7:**
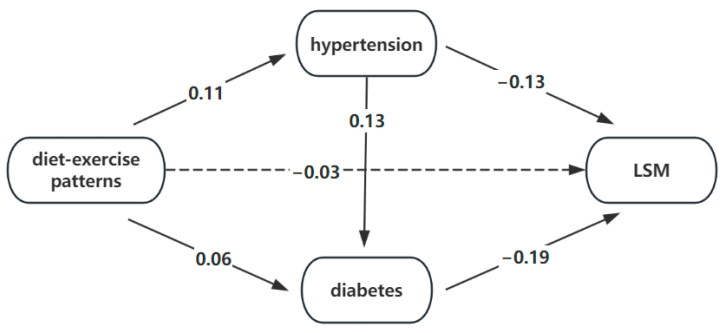
The medication effect of hypertension and diabetes.

In order to address the uncertainties arising from the results of the previous two sections, we utilized these associated diseases (hypertension, diabetes, anemia, high cholesterol levels) as mediating variables to examine the impact of diet–exercise patterns on liver cirrhosis. The results of the mediation analysis showed that two diseases, hypertension and diabetes, were important mediating factors between diet–exercise patterns and liver cirrhosis. Their mediating effects were, respectively, 24.48% (*p* < 0.001) and 19.51% (*p* < 0.001). The results of their chain mediating effect are shown in Figure 7.

## 4. Discussion

By utilizing a relatively new unsupervised machine learning method to analyze the sample population of the NHANES 2017-March 2020, we identified two diet–exercise patterns: “prudent pattern” and “dangerous pattern”. Of these, people in the “prudent pattern” tended to eat a variety of staple foods, eggs, meat, vegetables and fruits, were and less sedentary. People in the “dangerous pattern” ate more desserts, nuts, milk, meat, alcoholic beverages, and did more recreational activities. We then further demonstrated a strong link between diet–exercise habits, as indicated by these two diet–exercise patterns, and their risk of developing cirrhosis. This suggested that individuals following the “prudent pattern” had a lower risk.

### 4.1. Selection of Pattern Factors

In this study, diet items and exercise items were included to classify patterns affecting disease outcomes. Based on this, we analyzed the patterns and diseases. In previous studies, patterns typically included only dietary information. CORRAO et al. [38,39] conducted a principal component analysis on dietary data, proving the relationship between certain nutritional factors and the risk of LC. Guo et al. [40] also conducted a principal component analysis of dietary data, identifying two main dietary patterns. FARCHI et al. [14] conducted a cluster analysis on the sample population and divided it into four categories. These studies suggested that food intakes might be associated with the risk of chronic liver disease. Then, we considered that both diet items and exercise items had positive effects on multiple chronic diseases [41]. Exercise training has been shown to improve the health of patients with cirrhosis [42]. Therefore, we believed that it was necessary to incorporate exercise into the calculation of dietary patterns. In our results, the exercise factors do play an important role in the patterns, especially sedentary and recreational activities.

### 4.2. The Related Factors of Liver Cirrhosis

There is a large variation in the tendency of individuals to develop cirrhosis, which may be closely related to individual genetic factors and lifestyle habits. Previous studies have explored the association between cirrhosis and individual foods or nutrients. For instance, BRIDGES et al. [43] found a positive correlation between pork and beef and cirrhosis. The mechanism of the effect of protein, fiber and coffee on liver cirrhosis may be related to the duodenal microflora. Reducing the inflammatory potential of one’s diet may provide novel strategies to prevent the progression of cirrhosis [44,45]. In addition, the liver is a major organ responsible for zinc metabolism, and zinc deficiency may also alter liver cell function and immune response in inflammatory liver disease [2]. The protective and risk factors that these results point to are consistent with the items included in our prudent and dangerous patterns. Moreover, other sub-items in our patterns, particularly macro-nutrients and micro-nutrients, are likely to have significant effects on disease. Improving individual living habits, whether from the perspective of individual factors or from the level of overall dietary patterns, can have a positive impact on health conditions.

We further investigated the association between characteristics of the general population and cirrhosis. The results showed that low education, smoking, alcohol consumption and other factors are all related to the severity of cirrhosis progression. There are findings that support the causal protective effect of education on chronic liver disease [46,47]. There is evidence suggesting that smoking increases the risk of fibrosis progression and the development of hepatocellular carcinoma [48,49]. Also, individual differences in liver sensitivity to alcohol may influence the risk of developing this condition [49,50]. However, it has been suggested that women are more susceptible to the harmful effects of alcohol and have a higher risk of developing cirrhosis and related complications. Additionally, women also exhibit a higher rate of fibrosis progression after liver transplantation [51,52]. Our study suggests that women have a lower risk of cirrhosis than men, which is inconsistent with previous studies. This may be because many of the men in our sample had smoking and drinking habits.

### 4.3. Other Discussions about Disease Prevention

In summary, we obtained two diet–exercise patterns. These two patterns are not associated with CAPs but are significantly associated with LSMs. We believe that the combination of these items has little impact on early liver lesions, but is very useful for the disease outcome of patients with advanced liver lesions, especially cirrhosis. Lifestyle modifications targeting these combinations may not change disease development or exacerbation in patients with hepatic steatosis, but they may have a positive effect on the condition of patients with hepatic fibrosis. The results of our single-factor logistic regression analysis and multi-factor logistic regression analysis including demographic characteristics showed that the prudent pattern was a protective factor for LSMs. However, according to the multi-factor logistic regression analysis after adding hypertension, diabetes and other related diseases, the effect of the patterns on cirrhosis was not statistically significant. In order to determine the reason for this situation, we conducted a further mediation analysis and discovered that hypertension and diabetes were significant mediating factors between diet–exercise patterns and liver cirrhosis. Therefore, we discussed that the effect of the diet–exercise patterns was largely due to their impact on hypertension and diabetes, which led to the progression of liver cirrhosis. Thus, we suggest that adjusting diet and exercise, along with controlling blood pressure and glucose, can play a better role in preventing liver cirrhosis.

The main strength of our study was that this study is the first to address a specific target, population-wide U.S. diet–exercise patterns of cirrhosis risk. First of all, in the studies on chronic liver diseases, most diet studies have tended to focus on changes in the related traits of liver fat. However, our study added to the current knowledge on the impact of diet and exercise on liver cirrhosis, emphasizing the relationship between an unhealthy lifestyle and the possibility of increasing the degree of liver fibrosis and cirrhosis. We also incorporated exercise information and obtained more accurate analysis results on the living habits of the population. Secondly, in the study of dietary habits in liver cirrhosis, more studies are based on a priori pattern analysis or single-nutrient analysis, which often could obtain accurate and beneficial results. Of course, these results always cover what can be explained by posterior analyses. For example, whether a priori or a posteriori, we always obtain the conclusion that moderate vitamin D intake is likely to be good for health, which is often constant and consistent with our life experience. Nevertheless, we believe that a posteriori analysis—the statistical -partitioning of dietary patterns—is useful because it takes into account the relationship between food and nutrients, food interactions and nutrient interactions. For example, in the “prudent pattern” and “dangerous pattern” divided by the method of clustering of principal components, the intake of some vitamins is higher in the “prudent pattern” group, and the intake of others is higher in the “dangerous pattern” group. We cannot directly determine whether a certain vitamin is beneficial or harmful to the human body. This is because they interact with various other foods during ingestion. And how to make a better combination between various dietary factors and exercise factors, which is also the significance of posterior partition patterns. In the process of pattern division, different methods have different directivity and accuracy. We think that the method of this study is suitable for such analyses. The application of a relatively new method to analyze dietary patterns in cirrhosis is also one of the strengths of our study. This method overcomes the limitations of classical methods, which only focus on food itself and ignore individual living habits. Thirdly, we applied it to the data of the NHANES, which was a national sample survey in the United States. The research results of the NHANES could be radiated to the national population of the United States through the calculation of specific survey weights, which also made the research results more population-specific and made the conclusion credible. The two diet–exercise patterns that we obtained have confirmed an association with cirrhosis, and the differences in lifestyle habits between the two patterns can provide guidance on preventive diets for people at risk of cirrhosis. At the same time, these two patterns were also related to other diseases, such as diabetes and anemia, indicating that changing the dietary habits of the population can lead to more positive improvements in their health status. Finally, we conducted an effective mediation analysis of liver cirrhosis, which revealed the mediating role of diabetes and hypertension in the progression of liver cirrhosis. This may remind us of the relationship between diet and comorbidities.

The limitation of our study was that the selective elimination of the principal components may result in the loss of some original information from the food groups, potentially affecting the accuracy of the results. And we only briefly discussed the role of some related diseases in mediating the relationship between patterns and cirrhosis. The exploration of comorbidities between cirrhosis and related diseases, as well as the possible effects of diet–exercise patterns on these comorbidities, especially liver cancer, which may have a longitudinal relationship with the progression of cirrhosis, are also questions we need to consider for in-depth analysis in our upcoming studies. Another limitation of this study was the potential issue with data sources. We used the 24 h dietary recall data from the questionnaires in the NHANES database. Similarly, our physical activity data were also obtained from the recall interviews on the amount of physical activity performed in a week/day. However, there is inevitably some recall bias in the data obtained from these recall interviews. We have not been able to solve the problem. How to reduce the impact of these biases on lifestyle patterns and other research outcomes is also a topic that needs further research in the future.

## 5. Conclusions

The method of clustering of principal components describes the two main diet–exercise patterns associated with cirrhosis in the NHANES population: the “prudent pattern” and the “dangerous pattern”. The risk of disease in the “dangerous pattern” is relatively higher than that in the “prudent pattern”. The results of this study may help with preventive diets for people at risk of cirrhosis.

## Figures and Tables

**Table 1:** Grading criteria of hepatic steatosis and fibrosis.

CAP		LSM	
Degree of Steatosis	Grading Criteria (dB/m)	Degree of Fibrosis	Grading Criteria (kPa)
Absence of steatosis (S0)	<274	No fibrosis	<8.2
Mild steatosis (S1)	274~289	Significant fibrosis (F2)	8.2~9.6
Moderate steatosis (S2)	290~301	Severe fibrosis (F3)	9.7~13.5
Severe steatosis (S3)	≥302	Cirrhosis (F4)	≥13.6

**Table 2:** Total variance explained by principal components.

Principal Component	Eigenvalue	% Variance	% Cumulative
1	1.940680	0.067254	0.067254
2	1.328780	0.031530	0.098784
3	1.237584	0.027350	0.126134
4	1.218778	0.026525	0.152659

**Table 3:** The distribution of the sociodemographic characteristics of the study population across the two patterns.

Characteristics	Pattern 1 (%)	Pattern 2 (%)	*p* Value
Age			0.002
<30	3,241,828 (14.76)	35,007,500 (21.22)	
30–65	13,536,842 (61.56)	100,052,197 (60.63)	
>65	5,179,936 (23.59)	29,948,397 (18.15)	
Gender			<0.001
Male	9,193,537 (41.87)	83,202,734 (50.42)	
Female	12,765,069 (58.13)	81,805,359 (49.58)	
Race			<0.001
Mexican American	2,495,952 (11.37)	12,688,146 (7.69)	
Other Hispanic	2,499,944 (11.38)	11,002,573 (6.67)	
Non-Hispanic White	4,143,986 (18.87)	115,321,210 (69.89)	
Non-Hispanic Black	8,385,491 (38.19)	11,980,295 (7.26)	
Non-Hispanic Asian	3,344,518 (15.23)	7,116,489 (4.31)	
Other Race—Including Multi-Racial	1,088,714 (4.96)	6,899,379 (4.18)	
Education			<0.001
Less than high school	4,871,607 (22.19)	13,064,312 (7.92)	
High school graduate	5,370,959 (24.46)	45,301,154 (27.45)	
Some college or AA degree	7,028,360 (32.01)	51,257,818 (31.06)	
College graduate or above	4,687,680 (21.35)	55,384,809 (33.56)	
Marital Status			<0.001
Married/Living with partner	11,905,527 (54.22)	105,625,962 (64.01)	
Widowed/divorced/separated	5,605,759 (25.53)	27,685,874 (16.78)	
Never married	4,447,320 (20.25)	31,696,257 (19.21)	
BMI			0.093
<25	6,261,554 (28.52)	43,502,609 (26.36)	
~30	6,617,477 (30.14)	53,427,479 (32.38)	
~35	4,559,972 (20.77)	37,975,128 (23.01)	
~40	2,562,512 (11.67)	17,343,029 (10.51)	
>40	1,957,091 (8.91)	12,759,848 (7.73)	
Smoke			0.036
Yes	8,577,129 (39.06)	70,098,867 (42.48)	
No	13,381,476 (60.94)	94,909,226 (57.52)	
Drink			<0.001
Yes	19,474,976 (88.69)	155,278,680 (94.10)	
No	2,483,630 (11.31)	9,729,413 (5.90)	

**Table 4:** Factors associated with CAPs.

Characteristics	Simple OR (95%CI)	Stepwise (Part) OR (95%CI)	Stepwise (All) OR (95%CI)
Cluster characteristic			
Prudent pattern (reference = dangerous pattern)	1.04 (0.92–1.18)	1.04 (0.89–1.21)	-
Sociodemographic characteristics			
Age (continuous)	1.02 (1.01–1.02)	1.02 (1.02–1.03)	1.02 (1.01–1.02)
Gender (reference = male)	0.62 (0.51–0.93)	0.47 (0.40–0.57)	0.52 (0.44–0.62)
Race (reference = Mexican American)			
Other Hispanic	0.60 (0.47–0.76)	0.57 (0.43–0.76)	0.59 (0.45–0.76)
Non-Hispanic White	0.60 (0.46–0.71)	0.47 (0.37–0.59)	0.48 (0.37–0.61)
Non-Hispanic Black	0.39 (0.34–0.44)	0.25 (0.20–0.31)	0.23 (0.19–0.29)
Non-Hispanic Asian	0.49 (0.40–0.61)	0.99 (0.71–1.38)	0.91 (0.64–1.30)
Other Race—Including Multi-Racial	0.72 (0.50–1.04)	0.62 (0.39–0.99)	0.57 (0.34–0.95)
Education (reference = college graduate or above)			
Less than high school	1.44 (1.05–1.96)	0.99 (0.69–1.43)	-
High school graduate	1.68 (1.32–2.15)	1.30 (1.00–1.69)	1.23 (0.97–1.56)
Some college or AA degree	1.53 (1.30–1.79)	1.27 (1.04–1.57)	1.19 (0.98–1.45)
Marital Status (reference = married/living with Partner)			
Widowed/divorced/separated	0.78 (0.64–0.95)	0.72 (0.54–0.96)	0.68 (0.51–0.92)
Never married	0.54 (0.45–0.56)	0.66 (0.52–0.85)	0.67 (0.53–0.85)
BMI (continuous)	1.20 (1.18–1.23)	1.23 (1.20–1.25)	1.22 (1.19–1.25)
Smoke (reference = no)	1.21 (1.01–1.32)	1.12 (0.88–1.43)	1.13 (0.89–1.45)
Drink (reference = no)	1.17 (0.80–1.73)	1.62 (1.20–2.17)	1.61 (1.19–2.67)
Relative diseases			
Hypertension (reference = no)	2.76 (2.35–3.26)	-	1.49 (1.27–1.75)
Diabetes (reference = no)			
Yes	3.73 (2.86–4.86)	-	1.78 (1.30–2.43)
Borderline	2.59 (1.51–4.44)	-	1.23 (0.79–1.93)
High cholesterol level (reference = no)	1.63 (1.36–1.96)	-	1.05 (0.91–1.21)
Weak/failing kidneys (reference = no)	1.53 (0.93–2.50)	-	0.96 (0.58–1.61)
Anemia (reference = no)	0.81 (0.56–1.15)	-	0.68 (0.41–1.12)
Cancer (reference = no)	1.35 (1.02–1.77)	-	1.09 (0.81–1.47)
Hepatitis B (reference = no)	1.05 (0.52–2.14)	-	1.69 (1.03–2.78)
Hepatitis C (reference = no)	0.60 (0.27–1.34)	-	0.85 (0.42–1.71)

**Table 5:** Factors associated with LSMs.

Characteristics	Simple OR (95%CI)	Stepwise (Part) OR (95%CI)	Stepwise (All) OR (95%CI)
Cluster characteristic			
Prudent pattern (reference = dangerous pattern)	0.73 (0.59–0.93)	0.78 (0.56–0.99)	0.83 (0.62–1.10)
Sociodemographic characteristics			
Age (continuous)	1.02 (1.01–1.03)	1.02 (1.01–1.04)	1.02 (1.00–1.03)
Gender (reference = male)	0.5 (0.35–0.71)	0.35 (0.24–0.50)	0.42 (0.28–0.61)
Race (reference = Mexican American)			
Other Hispanic	0.89 (0.53–1.51)	0.87 (0.52–1.48)	0.89 (0.52–1.52)
Non-Hispanic White	0.87 (0.58–1.32)	0.89 (0.51–1.55)	0.86 (0.49–1.49)
Non-Hispanic Black	0.86 (0.60–1.24)	0.65 (0.42–1.02)	0.62 (0.39–1.00)
Non-Hispanic Asian	0.58 (0.32–1.04)	1.16 (0.60–2.25)	1.17 (0.58–2.34)
Other Race—Including Multi-Racial	0.95 (0.52–1.73)	0.87 (0.44–1.73)	0.87 (0.38–1.98)
Education (reference = college graduate or above)			
Less than high school	2.57 (1.72–3.82)	1.81 (1.08–3.02)	1.86 (1.06–3.11)
High school graduate	2.74 (1.74–4.33)	2.05 (1.17–3.58)	2.11 (1.18–3.77)
Some college or AA degree	1.74 (1.18–2.57)	1.32 (0.82–2.11)	1.45 (0.88–2.41)
Marital Status (reference = married/living with partner)			
Widowed/divorced/separated	1.02 (0.73–1.43)	0.98 (0.69–1.39)	0.95 (0.65–1.40)
Never married	0.71 (0.48–1.06)	0.88 (0.57–1.36)	0.85 (0.54–1.34)
BMI (continuous)	1.13 (1.11–1.15)	1.15 (1.12–1.17)	1.14 (1.12–1.17)
Smoke (reference = no)	1.45 (1.08–10.64)	-	1.10 (0.78–1.55)
Drink (reference = no)	1.20 (0.75–1.92)	-	1.32 (0.69–2.51)
Relative diseases			
Hypertension (reference = no)	2.79 (2.17–3.59)	-	1.37 (1.00–1.86)
Diabetes (reference = no)			
Yes	5.06 (3.68–6.96)	-	2.58 (1.81–3.68)
Borderline	4.11 (1.76–9.57)	-	2.00 (0.93–4.28)
High cholesterol level (reference = no)	1.32 (1.05–1.66)	-	1.42 (1.08–1.87)
Weak/failing kidneys (reference = no)	1.66 (0.91–3.04)	-	1.02 (0.51–2.02)
Anemia (reference = no)	0.81 (0.37–1.76)	-	0.68 (0.26–1.74)
Cancer (reference = no)	1.44 (0.88–2.35)	-	1.13 (0.75–1.69)
Hepatitis B (reference = no)	1.20 (0.53–2.73)	-	1.29 (0.41–4.04)
Hepatitis C (reference = no)	3.61 (1.77–7.33)	-	4.52 (2.19–9.36)

**Table 6:** The related indicators of liver cirrhosis with the two clusters.

Items	Prudent Pattern		Dangerous Pattern		*p* Value
	Mean	SD	Mean	SD	
ALT	21.490406	0.479526	23.106974	0.335490	<0.001
AST	21.804752	0.413814	21.897648	0.285381	0.016
GGT	34.597871	1.189280	28.551904	0.634683	0.001
ALP	79.184514	0.873206	74.194045	0.595263	<0.001
platelets	246.565500	2.190417	245.831812	2.075096	0.917
FIB4 score	0.552965	0.010495	0.459796	0.010593	<0.001
APRI score	0.249157	0.006719	0.242441	0.004654	0.060

**Table 7:** Relations of other diseases with the two clusters.

Diseases	Prudent Pattern	Dangerous Pattern	*p* Value
Hypertension			<0.001
Yes	8,376,303.32	49,404,887.08	
No	12,020,102.53	108,040,459.90	
Unclear	13,681.83	201,556.01	
Diabetes			<0.001
Yes	3,678,424.72	15,251,204.94	
No	16,044,882.26	138,976,678.90	
Borderline	686,780.70	3,373,390.36	
Unclear	0.00	45,628.83	
High cholesterol level			0.036
Yes	7,466,640.30	55,067,231.70	
No	12,799,540.30	102,234,088.60	
Unclear	143,907.10	345,582.70	
Weak/failing kidneys			0.066
Yes	845,002.44	4,406,153.99	
No	19,526,184.57	153,127,240.50	
Unclear	38,900.67	113,508.57	
Anemia			<0.001
Yes	1,253,862.56	5,136,849.99	
No	19,117,978.38	152,449,807.40	
Unclear	38,246.74	60,245.60	
Cancer			0.443
Yes	1,966,659.00	16,701,760.00	
No	18,434,680.00	140,919,600.00	
Unclear	8752.74	25,543.26	
Hepatitis B			0.600
Yes	282,751.01	1,777,892.48	
No	20,056,424.13	155,561,266.90	
Unclear	70,912.55	307,743.62	
Hepatitis C			0.417
Yes	364,224.26	2,253,468.24	
No	19,976,243.68	155,098,150.60	
Unclear	69,619.74	295,284.14	

## Data Availability

All relative original data are publicly available at www.cdc.gov/nchs/nhanes/, accessed on 5 September 2023.

## Supplementary Materials

The following supporting information can be downloaded at: https://www.mdpi.com/article/10.3390/nu16111617/s1, Figure S1: Flowchart of the study subjects; Figure S2: Score plots of first four principal components obtained after principal component analysis; Table S1: List of 59 food and activity categories; Table S2: Factor loadings for categories and four principal components; Table S3: Characteristics across two diet–exercise patterns; Table S4: Comparison of nutrient intakes between patterns.
