# Supplementary material for: The Association between Diet–Exercise Patterns and Cirrhosis: A Cross-Sectional Study from NHANES 2017-March 2020
Source: Nutrients. 2024 May 25;16(11):1617. doi: 10.3390/nu16111617 (PMC11174719; doi:10.3390/nu16111617)
Supplement: Supplementary file 1 [file nutrients-16-01617-s001.zip › nutrients-2982283-supplementary.pdf]

The association between diet-exercise patterns and cirrhosis: A cross-sectional study from NHANES 2017-March 2020

Supplementary Materials

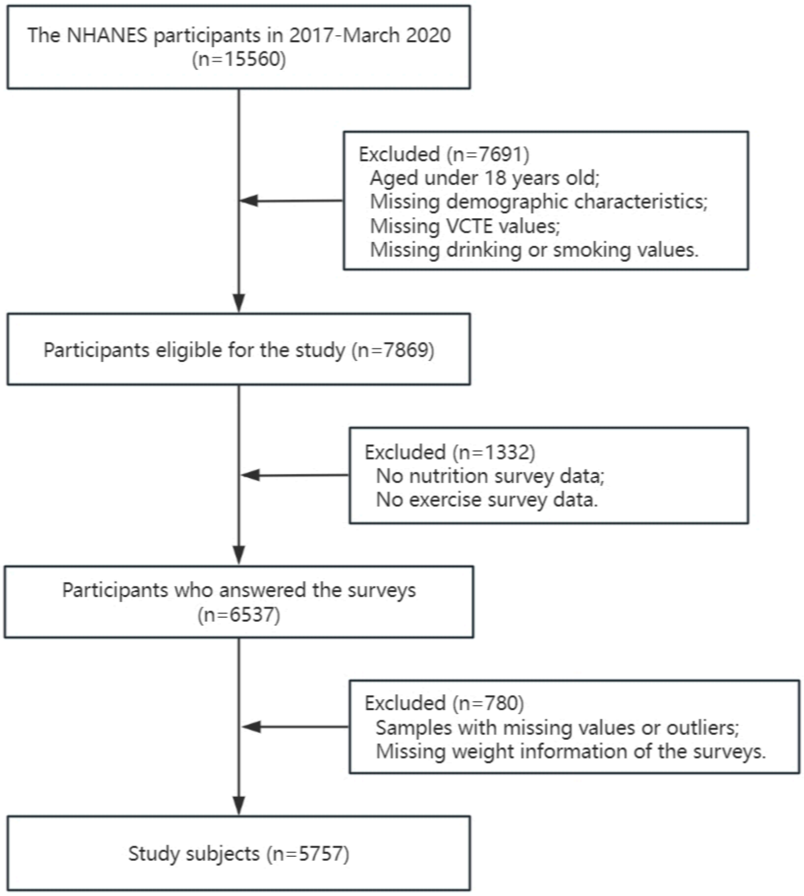

Figure S1. Flowchart of the study subjects

Table S1. List of 59 food and activity categories

Food Groups	Food Items
**1 Milk and Milk Products**	11 Milks, milk drinks, yogurts, infant formulas
	12 Creams and cream substitutes
	13 Milk desserts and sauces
	14 Cheeses
**2 Meat, Poultry, Fish, and Mixtures**	20 Meat
	21 Beef
	22 Pork
	23 Lamb, veal, game
	24 Poultry
	25 Organ meats, frankfurters, sausages, lunchmeats

	26 Fish, shellfish
	27 Meat, poultry, fish mixtures
	28 Frozen meals, soups, gravies
**3 Eggs**	31 Eggs
	32 Egg mixtures
	33 Egg substitutes
**4 Dry Beans, Peas, Other Legumes,Nuts, and Seeds**	41 Legumes
	42 Nuts, nut butters, nut mixtures
	43 Seeds and seed mixtures
	44 Carob products
**5 Grain Products**	50 Flour and dry mixes
	51 Yeast breads, rolls
	52 Quick breads
	53 Cakes, cookies, pies, pastries, bars
	54 Crackers, snack products
	55 Pancakes, waffles, French toast, other grain products
	56 Pastas, rice, cooked cereals
	57 Cereals, not cooked
	58 Grain mixtures, frozen meals, soups
	59 Meat substitutes
**6 Fruits**	61 Citrus fruits, juices
	62 Dried fruits
	63 Other fruits
	64 Fruit juices and nectars excluding citrus
	67 Fruits and juices baby food
**7 Vegetables**	71 White potatoes, starchy vegetables
	72 Dark-green vegetables
	73 Orange vegetables
	74 Tomatoes, tomato mixtures
	75 Other vegetables
	76 Vegetables and mixtures mostly vegetables baby food
	77 Vegetables with meat, poultry, fish
	78 Mixtures mostly vegetables without meat, poultry, fish
**8 Fats, Oils, and Salad Dressings**	81 Fats
	82 Oils
	83 Salad dressings
	89 ‘For use’ with a sandwich or vegetable
**9 Sugars, Sweets, and Beverages**	91 Sugars, sweets
	92 Nonalcoholic beverages
	93 Alcoholic beverages
	94 Noncarbonated water

	95 Formulated nutrition beverages, energy drinks, sports drink
	99 Used as an ingredient, not for coding
**10 Physical Activity**	101 Vigorous work activity
	102 Moderate work activity
	103 Walk or bicycle
	104 Vigorous recreational activity
	105 Moderate recreational activity
	106 Sedentary activity

Table S2. Factor loadings for categories and four principal components

Items	PC1	PC2	PC3	PC4
sedentary activity	-0.304973		-0.217177	
Noncarboted water	-0.273461		0.186921	
Nolcoholic beverages	-0.254224	-0.153065	-0.249584	
Moderate recreational activity	-0.206960		0.216941	
Salad dressings	-0.195236	0.207006		-0.191923
Other veg	-0.193362	0.214095		
Vigorous recreational activity	-0.191790		0.302069	
Moderate work activity	-0.185679	-0.408093	0.215955	
Yeast breads	-0.184995		-0.151567	
Other fruits	-0.184779	0.165117	0.210638	
Cheeses	-0.184523			
milk	-0.173572			0.539645
Crackers	-0.171453		-0.183282	
Sugars	-0.168609			
Tomatoes	-0.158302			
sausages	-0.157152			-0.208261
potatoes	-0.155815		-0.205145	
Pastas rice		0.197991	0.212457	
Grain mixtures		-0.222311		0.150598
Energy drinks		-0.153363		
Nuts		0.186835		
Dried fruits		0.205603		
Vigorous work activity		-0.449210	0.235236	
Walk or bicycle		-0.187694	0.195162	
Meat mixtures			-0.202705	
Legumes			0.194700	
Fats			-0.182403	
Orange veg			0.151125	
Mixtures veg			0.177110	
Cereals notcooked				0.560631

Poultry	-0.204759
Egg mixtures	-0.203077

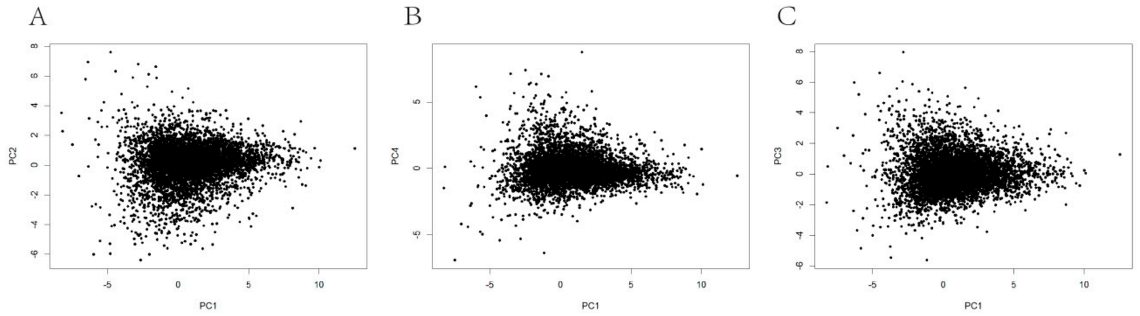

Figure S2. Score plots of the first four principal components obtained after principal component analysis.

Table S3. Characteristics across the two diet-exercise patterns

**Items**	**Pattern 1**		**Pattern 2**		***P value***
	mean	SD	mean	SD	
milk	79.929	3.6317	129.6363	5.8025	<0.001
gravies	20.3841	3.5844	21.4162	3.3887	0.194
Crackers	8.5968	0.5521	15.0128	0.6453	<0.001
Pancakes	3.9302	0.4669	4.5248	0.5491	0.577
Pastas rice	52.8272	2.9741	43.5973	3.3017	<0.001
Cereals uncooked	5.1676	0.4174	9.6298	0.5287	<0.001
Grain mixtures	139.283	7.1032	167.1475	6.0939	0.109
Fruit juices	21.5216	2.0645	21.9845	2.2197	0.376
Sugars	11.1082	0.6701	19.5604	0.8208	<0.001
Noncarbonated water	965.3367	23.1781	1332.1099	30.0235	<0.001
Cheeses	8.7724	0.5834	20.2794	1.5439	<0.001
Pork	8.6817	0.9465	11.0606	1.0165	0.016
Poultry	37.6871	2.5478	34.2877	1.6459	0.026
Yeast breads	25.1489	1.2964	33.804	1.4827	0.003
Tomatoes	13.6724	0.9468	27.0489	1.951	<0.001
Other veg	48.5266	2.2289	68.6444	4.2919	<0.001
Salad dressings	5.0278	0.3558	10.2252	0.9113	<0.001
sausages	8.4549	0.6144	15.7214	0.9123	<0.001
Cakes	26.4217	1.1058	34.4621	1.5188	<0.001
Other fruits	56.6858	2.9815	79.7175	4.3292	<0.001
potatoes	38.0864	1.914	52.146	2.26	0.03
Nonalcoholic beverages	589.4989	15.1936	868.7661	25.8814	<0.001
Energy drinks	24.1162	2.59	51.7935	4.499	<0.001
Meat mixtures	85.1103	3.3406	107.9511	4.7591	0.001

Dark green veg	9.1	0.7469	15.3993	2.0557	0.003
Quick breads	11.6329	1.3264	11.5324	0.9967	0.555
Alcoholic beverages	124.1619	7.7991	197.9718	12.8064	<0.001
Egg mixtures	12.3417	1.0672	16.452	1.1708	0.454
Legumes	17.2951	1.9571	19.4236	1.8932	0.275
cream	6.8493	0.4735	13.3438	1.0119	<0.001
Milk desserts	11.3006	1.0963	21.0558	1.3958	<0.001
Nuts	10.3488	0.9908	16.2262	2.0216	<0.001
Dried fruits	0.536	0.1175	1.402	0.2738	0.032
Fats	1.5934	0.1273	3.4455	0.2322	<0.001
Beef	9.462	1.1363	16.7268	1.7129	0.053
Fish	16.4819	1.9504	13.6985	1.0546	0.007
Eggs	7.9195	0.5451	12.1469	1.3931	0.012
Citrus fruits	32.6087	2.7264	33.2736	1.9921	0.588
Salad Foruse	4.1107	0.3152	6.8432	0.4623	<0.001
Fruits babyfood	0.1304	0.1315	0	0	0.332
Orange veg	4.6743	0.4345	10.1391	0.8066	<0.001
Seeds	0.3741	0.0899	0.8389	0.2455	0.023
sandwich	5.0975	0.6585	5.7506	0.6767	0.817
Oils	0.0473	0.0158	0.2383	0.0498	0.012
Mixtures veg	1.9184	0.5602	12.648	2.6703	<0.001
Lamb	0.285	0.0892	0.7157	0.1529	0.047
Egg substitutes	0.4387	0.1808	0.4896	0.2125	0.674
Meat	0	0	0.0073	0.0073	0.327
Meat substitutes	0	0	0.004	0.004	0.326
Veg with meat	0.2189	0.1538	0.0841	0.0615	0.344
Vigorous work activity	1183.6812	103.3181	2008.7431	134.0443	<0.001
Moderate work activity	969.9328	62.3282	1740.0484	81.4827	<0.001
Walk or bicycle	179.4142	20.1412	233.4919	21.9336	0.925
sedentary activity	298.5148	7.4013	359.499	5.004	<0.001
Vigorous recreational activity	240.7896	14.9473	647.6139	51.8126	<0.001
Moderate recreational activity	193.0697	8.2026	430.5634	16.2954	<0.001

Table S4. Comparison of nutrients intakes between patterns

Items	Pattern 1		Pattern 2		*P value*
	mean	SD	mean	SD	
Energy (kcal)	1684.3140	19.1769	2220.6453	17.5161	<0.001
Protein (gm)	62.4648	0.9300	84.4089	0.9177	<0.001
Carbohydrate (gm)	197.3266	2.5130	250.3510	2.6576	<0.001
Total sugars (gm)	83.1093	1.4388	107.7859	1.8562	<0.001
Dietary fiber (gm)	12.5827	0.3818	17.0854	0.3351	<0.001
Total fat (gm)	67.3267	0.8134	91.8636	0.8313	<0.001

Total saturated fatty acids (gm)	20.9371	0.3321	30.0841	0.3655	<0.001
Total monounsaturated fatty acids (gm)	23.3481	0.3038	31.3024	0.3854	<0.001
Total polyunsaturated fatty acids (gm)	16.2586	0.2152	21.3276	0.3090	<0.001
Cholesterol (mg)	247.3000	5.0234	326.8353	5.3604	<0.001
Vitamin E as alpha-tocopherol (mg)	6.9560	0.1283	9.7659	0.1663	<0.001
Added alpha-tocopherol (Vitamin E) (mg)	0.4796	0.0569	1.0528	0.0882	<0.001
Retinol (mcg)	269.3594	5.9153	429.3712	10.3960	<0.001
Vitamin A, RAE (mcg)	412.5130	8.2968	662.0790	13.6827	<0.001
Alpha-carotene (mcg)	260.3199	24.0440	386.7460	32.4181	<0.001
Beta-carotene (mcg)	1551.4498	82.4137	2555.9853	127.5133	<0.001
Beta-cryptoxanthin (mcg)	73.0784	4.1478	87.5797	5.6113	<0.001
Lycopene (mcg)	2994.3704	164.9088	5110.9316	220.0589	<0.001
Lutein + zeaxanthin (mcg)	1087.8334	68.3539	1717.2115	124.9255	<0.001
Thiamin (Vitamin B1) (mg)	1.2001	0.0178	1.6375	0.0191	<0.001
Riboflavin (Vitamin B2) (mg)	1.4155	0.0244	2.1893	0.0395	<0.001
Niacin (mg)	19.4352	0.2635	27.0105	0.4570	<0.001
Vitamin B6 (mg)	1.5112	0.0271	2.2516	0.0663	<0.001
Total folate (mcg)	283.1003	5.5845	385.8651	5.7525	<0.001
Folic acid (mcg)	122.4191	3.3056	164.6082	3.8775	<0.001
Food folate (mcg)	160.9732	3.5128	222.0777	4.1669	<0.001
Folate, DFE (mcg)	368.5714	7.5698	500.7214	7.8993	<0.001
Total choline (mg)	259.1744	4.3078	351.6048	4.3108	<0.001
Vitamin B12 (mcg)	3.4147	0.0906	5.0008	0.1375	<0.001
Added vitamin B12 (mcg)	0.4895	0.0460	1.0423	0.0860	<0.001
Vitamin C (mg)	60.7384	1.9586	78.8015	2.1622	<0.001
Vitamin D (D2 + D3) (mcg)	3.2630	0.1054	4.4986	0.1447	<0.001
Vitamin K (mcg)	90.6791	3.9737	132.9390	6.5425	<0.001
Calcium (mg)	674.4196	10.3424	990.6775	14.2009	<0.001
Phosphorus (mg)	1024.8972	16.3079	1429.8667	12.7610	<0.001
Magnesium (mg)	224.4518	4.1510	316.3351	3.6015	<0.001
Iron (mg)	10.4536	0.1707	14.3557	0.1601	<0.001
Zinc (mg)	8.0854	0.1854	11.2478	0.1402	<0.001
Copper (mg)	0.9010	0.0185	1.2592	0.0178	<0.001

Sodium (mg)	2722.2781	51.2216	3590.3178	42.1184	<0.001
Potassium (mg)	1928.6557	29.3656	2701.4774	27.4351	<0.001
Selenium (mcg)	88.8456	1.2920	117.4405	1.0281	<0.001
Caffeine (mg)	98.6466	4.2447	183.9477	6.6646	<0.001
Theobromine (mg)	22.3895	1.2288	42.4643	1.9744	<0.001
Alcohol (gm)	7.9491	0.5740	11.4611	0.6305	<0.001
Moisture (gm)	2223.7178	28.7701	3152.1997	40.3623	<0.001
